# Supplementary material for: Characterization of Long Non-Coding RNAs in the Bollworm, Helicoverpa zea, and Their Possible Role in Cry1Ac-Resistance
Source: Insects. 2021 Dec 22;13(1):12. doi: 10.3390/insects13010012 (PMC8779162; doi:10.3390/insects13010012)
Supplement: Supplementary file 1 [file insects-13-00012-s001.zip › insects-1520542-supplementary.pdf]

insects

MDPI

Supplementary Materials

Characterization of Long Non-Coding RNAs in the Bollworm, *Helicoverpa zea*, and Their Possible Role in Cry1Ac-Resistance

Roger D. Lawrie, Robert D. Mitchell III, Jean Marcel Deguenon, Loganathan Ponnusamy, Dominic Reisig, Alejandro Del Pozo-Valdivia, Ryan W. Kurtz and R. Michael Roe

Table S1. Increased expressed lncRNAs in Bt-resistant bollworms where a fold change could be calculated, including their fastq file ID (Fig. S2 numerical ID), gene annotation, magnitude of log2 fold increase, sequence length, BLASTn (NCBI) description, and fasta sequence BLASTn result details (E-value, % identity, and query coverage). The table was organized from greatest log2 fold increase to lowest.

Gene ID[a]	Gene Annotation (Accession #)[b]	Log2 Fold Increase[c]	BLASTn Result Description[d]	Sequence Length (bp)[e]	E-value[f]	% Identity[g]	Query Coverage (%)[h]
Hzea.12022 (1)	gi|1496272281|ref|XR_003401692.1|	4.8782	LOC113506107	1128	3.00E-84	85.58	36
Hzea.2506 (2)	gi|1496241937|ref|XR_003402053.1|	4.6556	LOC113508874	893	9.00E-55	80.89	25
Hzea.17450 (3)	gi|1199381004|ref|XR_002429340.1|	3.9043	LOC110372550	573	0.00E+00	95.78	98
Hzea.13715 (4)	gi|1199401540|ref|XR_002429826.1|	3.4978	LOC110380503	650	4.00E-169	94.42	76
Hzea.28004 (5)	gi|1199378765|ref|XR_002429309.1|	3.4416	LOC110371745	584	0.00E+00	95.95	72
Hzea.23814 (6)	gi|1199381581|ref|XR_002429343.1|	3.075	LOC110372708	358	2.00E-36	96.08	10
Hzea.30980 (7)	gi|1199404819|ref|XR_002429930.1|	3.0222	LOC110381881	854	8.00E-32	80	43
Hzea.21858 (8)	gi|1199370955|ref|XR_002430115.1|	2.9312	LOC110383908	1760	0.00E+00	90.3	70
Hzea.13175 (9)	gi|1199369172|ref|XR_002429735.1|	2.9255	LOC110379408	1193	0.00E+00	95.19	93
Hzea.604 (10)	gi|1199385412|ref|XR_002429411.1|	2.8328	LOC110374164	686	0.00E+00	98.83	95
Hzea.29875 (11)	gi|1199397344|ref|XR_002429683.1|	2.7328	LOC110378838	2247	0.00E+00	99.72	97
Hzea.20149 (12)	gi|1199396138|ref|XR_002429657.1|	2.5172	LOC110378368	507	0.00E+00	95.09	44
Hzea.12374 (13)	gi|1199385671|ref|XR_002429418.1|	2.4004	LOC110374252	1254	0.00E+00	90.38	45
Hzea.28389 (14)	gi|1199408314|ref|XR_002430068.1|	2.3533	LOC110383387	426	4.00E-77	94	24
Hzea.14942 (15)	gi|1200717987|ref|XR_002430221.1|	2.2453	LOC110384842	1264	1.00E-77	84.97	37
Hzea.16728 (16)	gi|1199408095|ref|XR_002430048.1|	2.1086	LOC110383295	576	0.00E+00	90.39	97
Hzea.2954 (17)	gi|1199406738|ref|XR_002429977.1|	2.0091	LOC110382662	364	1.00E-176	98.07	42
Hzea.26641 (18)	gi|1199385671|ref|XR_002429418.1|	1.9793	LOC110374252	1254	0.00E+00	90.72	39
Hzea.21239 (19)	gi|1199406154|ref|XR_002429966.1|	1.9431	LOC110382424	1833	0.00E+00	92.89	94
Hzea.20530 (20)	gi|1199384114|ref|XR_002429384.1|	1.8871	LOC110373699	1934	0.00E+00	98.96	96
Hzea.2658 (21)	gi|1199401627|ref|XR_002429835.1|	1.8262	LOC110380550	1351	0.00E+00	91.96	54
Hzea.7449 (22)	gi|1199399535|ref|XR_002429761.1|	1.8078	LOC110379665	1839	0.00E+00	98.36	100
Hzea.24315 (23)	gi|1199406738|ref|XR_002429977.1|	1.6599	LOC110382662	364	2.00E-176	98.08	27
Hzea.5298 (24)	gi|1199398266|ref|XR_002429709.1|	1.6476	LOC110379203	967	0.00E+00	94.94	77
Hzea.10028 (25)	gi|1199386863|ref|XR_002429444.1|	1.644	LOC110374708	522	0.00E+00	94.33	92
Hzea.12850 (26)	gi|1199398466|ref|XR_002429719.1|	1.5387	LOC110379254	3058	0.00E+00	93.57	41
Hzea.31031 (27)	gi|1199369172|ref|XR_002429735.1|	1.4668	LOC110379408	1193	0.00E+00	94.47	100
Hzea.29569 (28)	gi|1199389586|ref|XR_002429504.1|	1.4428	LOC110375772	794	0.00E+00	95.63	30
Hzea.2296 (29)	gi|1199381581|ref|XR_002429343.1|	1.3909	LOC110372708	358	8.00E-121	98.05	23
Hzea.17312 (30)	gi|1496283280|ref|XR_003400832.1|	1.3259	LOC113496559	3955	2.00E-45	92.75	4
Hzea.18336 (31)	gi|1199390745|ref|XR_002429524.1|	1.325	LOC110376247	1010	0.00E+00	96.84	99

<table>
<tr><td>Hzea.9709 (32)</td><td>gi|1199378738|ref|XR_002429307.1|</td><td>1.2877</td><td>LOC110371734</td><td>345</td><td>5.00E-152</td><td>95.4</td><td>88</td></tr>
<tr><td>Hzea.22246 (33)</td><td>gi|1199400576|ref|XR_002429791.1|</td><td>1.2241</td><td>LOC110380105</td><td>446</td><td>2.00E-173</td><td>91.78</td><td>78</td></tr>
<tr><td>Hzea.13913 (34)</td><td>gi|1199383324|ref|XR_002429366.1|</td><td>0.995</td><td>LOC110373344</td><td>402</td><td>0.00E+00</td><td>95.77</td><td>27</td></tr>
<tr><td>Hzea.15201 (35)</td><td>gi|1199372467|ref|XR_002430136.1|</td><td>0.9846</td><td>LOC110384418</td><td>353</td><td>2.00E-113</td><td>93.1</td><td>54</td></tr>
<tr><td>Hzea.30500 (36)</td><td>gi|1199366745|ref|XR_002429227.1|</td><td>0.9604</td><td>LOC110370099</td><td>1290</td><td>0.00E+00</td><td>96.73</td><td>76</td></tr>
<tr><td>Hzea.16487 (37)</td><td>gi|1200727923|ref|XR_001139805.3|</td><td>0.9318</td><td>LOC105841990</td><td>1689</td><td>2.00E-167</td><td>83.53</td><td>45</td></tr>
<tr><td>Hzea.31235 (38)</td><td>gi|1199370720|ref|XR_002430110.1|</td><td>0.9309</td><td>LOC110383825</td><td>745</td><td>2.00E-98</td><td>82.23</td><td>65</td></tr>
<tr><td>Hzea.19358 (39)</td><td>gi|1199383525|ref|XR_002429368.1|</td><td>0.8859</td><td>LOC110373445</td><td>6548</td><td>0.00E+00</td><td>88.73</td><td>73</td></tr>
<tr><td>Hzea.18335 (40)</td><td>gi|1199390744|ref|XR_002429523.1|</td><td>0.8859</td><td>LOC110376246</td><td>1288</td><td>0.00E+00</td><td>96.9</td><td>76</td></tr>
<tr><td>Hzea.3960 (41)</td><td>gi|1199406106|ref|XR_002429964.1|</td><td>0.8819</td><td>LOC110382392</td><td>2574</td><td>0.00E+00</td><td>95.2</td><td>90</td></tr>
<tr><td>Hzea.30284 (42)</td><td>gi|1199384299|ref|XR_002429388.1|</td><td>0.8088</td><td>LOC110373788</td><td>528</td><td>0.00E+00</td><td>99.05</td><td>37</td></tr>
<tr><td>Hzea.26682 (43)</td><td>gi|1199389560|ref|XR_002429501.1|</td><td>0.7866</td><td>LOC110375761</td><td>449</td><td>0.00E+00</td><td>98</td><td>78</td></tr>
<tr><td>Hzea.5967 (44)</td><td>gi|1199398055|ref|XR_002429700.1|</td><td>0.7861</td><td>LOC110379115</td><td>3649</td><td>0.00E+00</td><td>97.48</td><td>55</td></tr>
<tr><td>Hzea.32166 (45)</td><td>gi|1199395541|ref|XR_002429643.1|</td><td>0.7447</td><td>LOC110378136</td><td>418</td><td>0.00E+00</td><td>97.42</td><td>88</td></tr>
<tr><td>Hzea.9647 (46)</td><td>gi|1199380627|ref|XR_002429335.1|</td><td>0.7022</td><td>LOC110372433</td><td>913</td><td>0.00E+00</td><td>92.94</td><td>75</td></tr>
<tr><td>Hzea.28852 (47)</td><td>gi|1199403501|ref|XR_002429876.1|</td><td>0.6566</td><td>LOC110381286</td><td>544</td><td>2.00E-143</td><td>96.81</td><td>59</td></tr>
<tr><td>Hzea.11610 (48)</td><td>gi|1199380589|ref|XR_002429333.1|</td><td>0.6451</td><td>LOC110372421</td><td>1125</td><td>0.00E+00</td><td>84.05</td><td>21</td></tr>
<tr><td>Hzea.20367 (49)</td><td>gi|1199398907|ref|XR_002429736.1|</td><td>0.6257</td><td>LOC110379414</td><td>297</td><td>2.00E-146</td><td>98.99</td><td>77</td></tr>
<tr><td>Hzea.646 (50)</td><td>gi|1199397854|ref|XR_002429694.1|</td><td>0.6096</td><td>LOC110379030</td><td>387</td><td>0.00E+00</td><td>98.46</td><td>83</td></tr>
<tr><td>Hzea.20502 (51)</td><td>gi|1199405118|ref|XR_002429943.1|</td><td>0.5774</td><td>LOC110382011</td><td>1835</td><td>0.00E+00</td><td>90.67</td><td>68</td></tr>
<tr><td>Hzea.23711 (52)</td><td>gi|1496280423|ref|XR_003400740.1|</td><td>0.5217</td><td>LOC113495534</td><td>6719</td><td>6.00E-135</td><td>83.86</td><td>15</td></tr>
<tr><td>Hzea.22755 (53)</td><td>gi|1199407247|ref|XR_002430000.1|</td><td>0.4906</td><td>LOC110382901</td><td>936</td><td>0.00E+00</td><td>95.05</td><td>33</td></tr>
<tr><td>Hzea.24620 (54)</td><td>gi|1199382262|ref|XR_002429351.1|</td><td>0.4682</td><td>LOC110372972</td><td>4231</td><td>0.00E+00</td><td>98.39</td><td>100</td></tr>
<tr><td>Hzea.9489 (55)</td><td>gi|1199408314|ref|XR_002430068.1|</td><td>0.4173</td><td>LOC110383387</td><td>426</td><td>1.00E-87</td><td>94.91</td><td>19</td></tr>
<tr><td>Hzea.8708 (56)</td><td>gi|1199387699|ref|XR_002429464.1|</td><td>0.4137</td><td>LOC110375033</td><td>3217</td><td>0.00E+00</td><td>96.3</td><td>93</td></tr>
<tr><td>Hzea.17033 (57)</td><td>gi|1274103780|ref|XR_002696594.1|</td><td>0.4135</td><td>LOC111347786</td><td>1072</td><td>4.00E-04</td><td>94.74</td><td>2</td></tr>
<tr><td>Hzea.8067 (58)</td><td>gi|1199389764|ref|XR_002429506.1|</td><td>0.3767</td><td>LOC110375841</td><td>764</td><td>0.00E+00</td><td>98.17</td><td>90</td></tr>
</table>

[a] Gene number corresponds to sequence number in fastq files. Number in parentheses corresponds to lncRNA number outlined in Fig. S2 x-axis; [b] Gene annotations are the database accession numbers for each sequence (ncbi); [c] log2 fold increase indicates the magnitude of increase of expression in the Bt-resistant strain; [d] Description provides the LOC number for the top ncbi BLASTn match; [e] Sequence length indicates the number of base-pairs in each lncRNA; [f] LncRNA fastq sequence BLASTn e-value result; [g] LncRNA fastq file BLASTn percent identity result; [h] LncRNA fastq sequence BLASTn query coverage result.

Table S2. Decreased expressed lncRNAs in Bt-resistant bollworms where a fold change could be calculated, including their fastq file ID (Fig. S2 numerical ID), gene annotation, magnitude of log2 fold decrease, sequence length, BLASTn (NCBI) description, and fasta sequence BLASTn result details (E-value, % identity, and query coverage). The table was organized from greatest log2 fold decrease to lowest.

<table>
<tr><th>Gene ID[a]</th><th>Gene Annotation (Accession #)[b]</th><th>Log2 Fold Decrease[c]</th><th>BLASTn Result Description[d]</th><th>Sequence Length (bp)[e]</th><th>E-value[f]</th><th>% Identity[g]</th><th>Query Coverage (%)[h]</th></tr>
<tr><td>Hzea.26537 (59)</td><td>gi|1199367711|ref|XR_002429389.1|</td><td>4.40755</td><td>LOC110373805</td><td>659</td><td>7.00E-173</td><td>92.97</td><td>28</td></tr>
<tr><td>Hzea.14205 (60)</td><td>gi|1199383740|ref|XR_002429373.1|</td><td>3.68166</td><td>LOC110373534</td><td>4491</td><td>3.00E-30</td><td>85.62</td><td>33</td></tr>
<tr><td>Hzea.3574 (61)</td><td>gi|1199406738|ref|XR_002429977.1|</td><td>3.46104</td><td>LOC110382662</td><td>364</td><td>2.00E-160</td><td>95.33</td><td>25</td></tr>
<tr><td>Hzea.17384 (62)</td><td>gi|1199408414|ref|XR_002430074.1|</td><td>3.05139</td><td>LOC110383440</td><td>635</td><td>3.00E-96</td><td>90.55</td><td>47</td></tr>
<tr><td>Hzea.20392 (63)</td><td>gi|1199373558|ref|XR_002429212.1|</td><td>2.83539</td><td>LOC110369725</td><td>947</td><td>0.00E+00</td><td>96.23</td><td>98</td></tr>
<tr><td>Hzea.13495 (64)</td><td>gi|1199369844|ref|XR_002429941.1|</td><td>2.48753</td><td>LOC110382000</td><td>414</td><td>2.00E-112</td><td>96.12</td><td>9</td></tr>
<tr><td>Hzea.10752 (65)</td><td>gi|1199370723|ref|XR_002430111.1|</td><td>1.68549</td><td>LOC110383827</td><td>1221</td><td>5.00E-175</td><td>85.81</td><td>42</td></tr>
<tr><td>Hzea.353 (66)</td><td>gi|1199378516|ref|XR_002429294.1|</td><td>1.54594</td><td>LOC110371636</td><td>670</td><td>8.00E-166</td><td>97.69</td><td>21</td></tr>
<tr><td>Hzea.31686 (67)</td><td>gi|1199391104|ref|XR_002429541.1|</td><td>1.48642</td><td>LOC110376383</td><td>347</td><td>1.00E-159</td><td>98.18</td><td>76</td></tr>
<tr><td>Hzea.28810 (68)</td><td>gi|1199371816|ref|XR_002430124.1|</td><td>1.43915</td><td>LOC110384191</td><td>325</td><td>4.00E-149</td><td>96.62</td><td>61</td></tr>
<tr><td>Hzea.29346 (69)</td><td>gi|1199374618|ref|XR_002429228.1|</td><td>1.34914</td><td>LOC110370109</td><td>552</td><td>0.00E+00</td><td>96.9</td><td>72</td></tr>
</table>

Hzea.4465 (70)	gi|1199374858|ref|XR_002429236.1|	1.21459	LOC110370222	375	7.00E-172	96.55	73
Hzea.9557 (71)	gi|1199393773|ref|XR_002429605.1|	1.20283	LOC110377404	327	2.00E-132	93.13	61
Hzea.22455 (72)	gi|1199398932|ref|XR_002429739.1|	0.982008	LOC110379429	714	0.00E+00	91.97	78
Hzea.23046 (73)	gi|1199371635|ref|XR_002430121.1|	0.937912	LOC110384132	611	0.00E+00	94.03	91
Hzea.12925 (74)	gi|1199404527|ref|XR_002429917.1|	0.902824	LOC110381748	1117	0.00E+00	91.87	94
Hzea.12351 (75)	gi|1199391501|ref|XR_002429554.1|	0.747984	LOC110376565	1450	3.00E-100	78.78	36
Hzea.4323 (76)	gi|1199382408|ref|XR_002429355.1|	0.701294	LOC110373015	1886	4.00E-19	87.38	6
Hzea.9846 (77)	gi|1199408413|ref|XR_002430073.1|	0.669071	LOC110383440	337	5.00E-140	94.38	13
Hzea.7487 (78)	gi|1199391858|ref|XR_002429559.1|	0.635993	LOC110376693	752	0.00E+00	93.96	88
Hzea.4468 (79)	gi|1199374635|ref|XR_002429229.1|	0.575654	LOC110370116	364	3.00E-179	98.35	90
Hzea.26043 (80)	gi|1199369334|ref|XR_002429787.1|	0.561559	LOC110380046	423	0.00E+00	95.52	93
Hzea.26045 (81)	gi|1199369284|ref|XR_002429773.1|	0.449016	LOC110379830	1820	0.00E+00	96.54	83
Hzea.4909 (82)	gi|1199389298|ref|XR_002429490.1|	0.441255	LOC110375656	1081	0.00E+00	97.78	41

[a] Gene number corresponds to sequence number in fastq files. Number in parentheses corresponds to lncRNA number outlined in Figure S2 x-axis; [b] Gene annotations are the database accession numbers for each sequence (ncbi); [c] log2 fold decrease indicates the magnitude of decrease of expression in the Bt-resistant strain; [d] Description provides the LOC number for the top ncbi BLASTn match; [e] Sequence length indicates the number of base-pairs in each lncRNA; [f] LncRNA fastq sequence BLASTn e-value result; [g] LncRNA fastq file BLASTn percent identity result; [h] LncRNA fastq sequence BLASTn query coverage result.

Table S3. LncRNAs found only in the Bt-resistant or Bt-susceptible bollworms including their fastq file ID (Fig. S2 numerical ID), gene annotation, strain of bollworm, sequence length, BLASTn (NCBI) description, and fasta sequence BLASTn result details (E-value, % identity, query coverage). This table was organized by strain (either Bt-susceptible or Bt-resistant).

	Gene ID[a]	Gene Annotation (Accession #)[b]	BLASTn Result Description[c]	Sequence Length (bp)[d]	E-value[e]	% Identity[f]	Query Coverage (%)[g]
Only in Resistant	Hzea.7318 (83)	gi|1199406283|ref|XR_002429971.1	LOC110382476	414	0	97.34	32
	Hzea.7825 (84)	gi|1199406761|ref|XR_002429982.1	LOC110382674	592	0	97.68	99
	Hzea.14864 (85)	gi|1199406977|ref|XR_002429992.1	LOC110382777	864	2.00E-50	99.15	11
	Hzea.3353 (86)	gi|1199383525|ref|XR_002429368.1	LOC110373445	6548	5.00E-51	96.21	16
	Hzea.1819 (87)	gi|1199398970|ref|XR_002429741.1	LOC110379447	377	9.00E-37	90.48	22
	Hzea.14022 (88)	gi|1199395149|ref|XR_002429636.1	LOC110377983	535	0	97.09	32
	Hzea.9756 (89)	gi|1199387541|ref|XR_002429457.1	LOC110374968	346	8.00E-114	94.51	99
	Hzea.27283 (90)	gi|1199367964|ref|XR_002429443.1	LOC110374671	647	5.00E-156	97.85	100
	Hzea.203 (91)	gi|1199378765|ref|XR_002429309.1	LOC110371745	584	3.00E-79	98.84	56
	Hzea.29688 (92)	gi|1199369338|ref|XR_002429789.1	LOC110380067	753	3.00E-57	91.72	81
Only in Susceptible	Hzea.28800 (93)	gi|1496279844|ref|XR_003400705.1	LOC113495312	2497	2.00E-166	89.89	99
	Hzea.4268 (94)	gi|1199385884|ref|XR_002429423.1	LOC110374339	334	6.00E-150	96.91	31
	Hzea.31700 (95)	gi|1199391125|ref|XR_002429544.1	LOC110376393	465	1.00E-73	91.08	61
	Hzea.19053 (96)	gi|1199408314|ref|XR_002430068.1	LOC110383387	426	1.00E-152	95.94	92

[a] Gene number corresponds to sequence number in fastq files. Numbers in parentheses correspond to lncRNA number outlined in Figure S2 x-axis; [b] Gene annotations are the database accession numbers for each sequence (ncbi); [c] Description provides the LOC number for the top ncbi BLASTn matc; [d] Sequence length indicates the number of base-pairs in each lncRNA; [e] LncRNA fastq sequence BLASTn e-value result; [f] LncRNA fastq file BLASTn percent identity result; [g] LncRNA fastq sequence BLASTn query coverage result.

Table S4. All NCBI BLAST alignments conducted while searching for potential pseudogenes. The top 5 up- and top 5 down-regulated lncRNAs were aligned to the highest log2 fold change (up- and down-regulated) coding-genes for 5 different categories of genes with functions associated with Bt-resistance in *H. zea*. Table depicts from left to right lncRNA direction of expression, fasta ID and accession number, coding-gene direction of expression, coding gene fasta ID and annotation, presence of sequence alignment, E-value, percent identity, and query coverage.

lncRNA direction of expression[a]	lncRNA ID[b]	Coding-gene direction of expression[c]	Coding Gene ID[d]	Sequence Alignment[e]	E-value[f]	% Identity[g]	Query Coverage (%)[h]

**Up-regulated**	Hzea.12022 (*T. ni* uncharacterized LOC113506107)	**Up-regulated**	Hzea.7824 (serine protease snake-like)	No significant alignment			
			Hzea.18477 (AY2 tetraspanin 1)	No significant alignment			
			Hzea.4257 (trypsin 3A1-like)	No significant alignment			
			Hzea.30068 (beta-secretase 1-like)	No significant alignment			
			Hzea.11178 (JP126 mutant cadherin)	No significant alignment			
		**Down-regulated**	Hzea.15356 (serine protease snake-like)	No significant alignment			
			Hzea.2673 (AY2 tetraspanin 1)	No significant alignment			
			Hzea.17647 (beta-secretase 1-like)	No significant alignment			
			Hzea.15893 (trypsin 5G1-like)	No significant alignment			
			Hzea.20 (XJ-r15 cadherin)	No significant alignment			
	Hzea.2506 (*T. ni* uncharacterized LOC113508874)	**Up-regulated**	Hzea.7824 (serine protease snake-like)	No significant alignment			
			Hzea.18477 (AY2 tetraspanin 1)		0.048	93.75	1
			Hzea.4257 (trypsin 3A1-like)	No significant alignment			
			Hzea.30068 (beta-secretase 1-like)	No significant alignment			
			Hzea.11178 (JP126 mutant cadherin)	No significant alignment			
		**Down-regulated**	Hzea.15356 (serine protease snake-like)	No significant alignment			
			Hzea.2673 (AY2 tetraspanin 1)	No significant alignment			
			Hzea.17647 (beta-secretase 1-like)	No significant alignment			

		Hzea.15893 (trypsin 5G1-like)	No significant alignment
		Hzea.20 (XJ-r15 cadherin)	No significant alignment
Hzea.17450 (*H. armigera* uncharacterized LOC110372550)	**Up-regulated**	Hzea.7824 (serine protease snake-like)	No significant alignment
		Hzea.18477 (AY2 tetraspanin 1)	No significant alignment
		Hzea.4257 (trypsin 3A1-like)	No significant alignment
		Hzea.30068 (beta-secretase 1-like)	No significant alignment
		Hzea.11178 (JP126 mutant cadherin)	No significant alignment
	**Down-regulated**	Hzea.15356 (serine protease snake-like)	No significant alignment
		Hzea.2673 (AY2 tetraspanin 1)	No significant alignment
		Hzea.17647 (beta-secretase 1-like)	No significant alignment
		Hzea.15893 (trypsin 5G1-like)	No significant alignment
		Hzea.20 (XJ-r15 cadherin)	No significant alignment
Hzea.13715 (*H. armigera* uncharacterized LOC110380503)	**Up-regulated**	Hzea.7824 (serine protease snake-like)	No significant alignment
		Hzea.18477 (AY2 tetraspanin 1)	No significant alignment
		Hzea.4257 (trypsin 3A1-like)	No significant alignment
		Hzea.30068 (beta-secretase 1-like)	No significant alignment
		Hzea.11178 (JP126 mutant cadherin)	No significant alignment
	**Down-regulated**	Hzea.15356 (serine protease snake-like)	No significant alignment
		Hzea.2673 (AY2 tetraspanin 1)	No significant alignment

			Hzea.17647 (beta-secretase 1-like)	No significant alignment			
			Hzea.15893 (trypsin 5G1-like)	No significant alignment			
			Hzea.20 (XJ-r15 cadherin)	No significant alignment			
	Hzea.28004 (*H. armigera* uncharacterized LOC110371745)	**Up-regulated**	Hzea.7824 (serine protease snake-like)	No significant alignment			
			Hzea.18477 (AY2 tetraspanin 1)	No significant alignment			
			Hzea.4257 (trypsin 3A1-like)	No significant alignment			
			Hzea.30068 (beta-secretase 1-like)	No significant alignment			
			Hzea.11178 (JP126 mutant cadherin)	No significant alignment			
		**Down-regulated**	Hzea.15356 (serine protease snake-like)	No significant alignment			
			Hzea.2673 (AY2 tetraspanin 1)	No significant alignment			
			Hzea.17647 (beta-secretase 1-like)	No significant alignment			
			Hzea.15893 (trypsin 5G1-like)	No significant alignment			
			Hzea.20 (XJ-r15 cadherin)	No significant alignment			
**Down-regulated**	Hzea.26537 (*H. armigera* uncharacterized LOC110373805)	**Up-regulated**	Hzea.7824 (serine protease snake-like)	No significant alignment			
			Hzea.18477 (AY2 tetraspanin 1)		0.027	94.12	0
			Hzea.4257 (trypsin 3A1-like)	No significant alignment			
			Hzea.30068 (beta-secretase 1-like)	No significant alignment			
			Hzea.11178 (JP126 mutant cadherin)	No significant alignment			
		**Down-regulated**	Hzea.15356 (serine protease snake-like)	No significant alignment			

		Hzea.2673 (AY2 tetraspanin 1)	No significant alignment				
		Hzea.17647 (beta-secretase 1-like)	No significant alignment				
		Hzea.15893 (trypsin 5G1-like)	No significant alignment				
		Hzea.20 (XJ-r15 cadherin)	No significant alignment				
Hzea.14205 (*H. armigera* uncharacterized LOC110373534)	**Up-regulated**	Hzea.7824 (serine protease snake-like)	No significant alignment				
		Hzea.18477 (AY2 tetraspanin 1)	No significant alignment				
		Hzea.4257 (trypsin 3A1-like)	No significant alignment				
		Hzea.30068 (beta-secretase 1-like)	No significant alignment				
		Hzea.11178 (JP126 mutant cadherin)	No significant alignment				
	**Down-regulated**	Hzea.15356 (serine protease snake-like)	No significant alignment				
		Hzea.2673 (AY2 tetraspanin 1)	No significant alignment				
		Hzea.17647 (beta-secretase 1-like)	No significant alignment				
		Hzea.15893 (trypsin 5G1-like)	No significant alignment				
		Hzea.20 (XJ-r15 cadherin)	No significant alignment				
Hzea.3574 (*H. armigera* uncharacterized LOC110382662)	**Up-regulated**	Hzea.7824 (serine protease snake-like)	No significant alignment				
		Hzea.18477 (AY2 tetraspanin 1)		4.00E-04	85.19	1	
		Hzea.4257 (trypsin 3A1-like)		0.018	94.12	1	
		Hzea.30068 (beta-secretase 1-like)	No significant alignment				
		Hzea.11178 (JP126 mutant cadherin)	No significant alignment				

		**Down-regulated**	Hzea.15356 (serine protease snake-like)	No significant alignment			
			Hzea.2673 (AY2 tetraspanin 1)		0.002	84.62	3
			Hzea.17647 (beta-secretase 1-like)	No significant alignment			
			Hzea.15893 (trypsin 5G1-like)	No significant alignment			
			Hzea.20 (XJ-r15 cadherin)		0.005	94.44	2
	Hzea.17384 (*H. armigera* uncharacterized LOC110383440)	**Up-regulated**	Hzea.7824 (serine protease snake-like)	No significant alignment			
			Hzea.18477 (AY2 tetraspanin 1)	No significant alignment			
			Hzea.4257 (trypsin 3A1-like)	No significant alignment			
			Hzea.30068 (beta-secretase 1-like)	No significant alignment			
			Hzea.11178 (JP126 mutant cadherin)	No significant alignment			
		**Down-regulated**	Hzea.15356 (serine protease snake-like)	No significant alignment			
			Hzea.2673 (AY2 tetraspanin 1)	No significant alignment			
			Hzea.17647 (beta-secretase 1-like)	No significant alignment			
			Hzea.15893 (trypsin 5G1-like)	No significant alignment			
			Hzea.20 (XJ-r15 cadherin)	No significant alignment			
	Hzea.20392 (*H. armigera* uncharacterized LOC110369725)	**Up-regulated**	Hzea.7824 (serine protease snake-like)	No significant alignment			
			Hzea.18477 (AY2 tetraspanin 1)	No significant alignment			
			Hzea.4257 (trypsin 3A1-like)	No significant alignment			
			Hzea.30068 (beta-secretase 1-like)		0.033	100	1

			Hzea.11178 (JP126 mutant cadherin)	No significant alignment			
		**Down-regulated**	Hzea.15356 (serine protease snake-like)	No significant alignment			
			Hzea.2673 (AY2 tetraspanin 1)	No significant alignment			
			Hzea.17647 (beta-secretase 1-like)	No significant alignment			
			Hzea.15893 (trypsin 5G1-like)	No significant alignment			
			**Hzea.20 (XJ-r15 cadherin)**		**0**	**99.07**	**81**

[a] Indicates the direction of expression for the lncRNA; [b] Indicates the fasta ID and the NCBI accession number for the lncRNA; [c] Indicates coding-gene direction of expression; [d] Indicates the coding-gene fasta ID and annotation ID; [e] Indicates whether the lncRNA and coding-gene had ncbi BLAST alignments; [f] NCBI assigned E-value for lncRNA to coding-gene alignment; [g] NCBI assigned percent identity for lncRNA to coding-gene alignment; [h] NCBI assigned query coverage for lncRNA to coding-gene alignment.

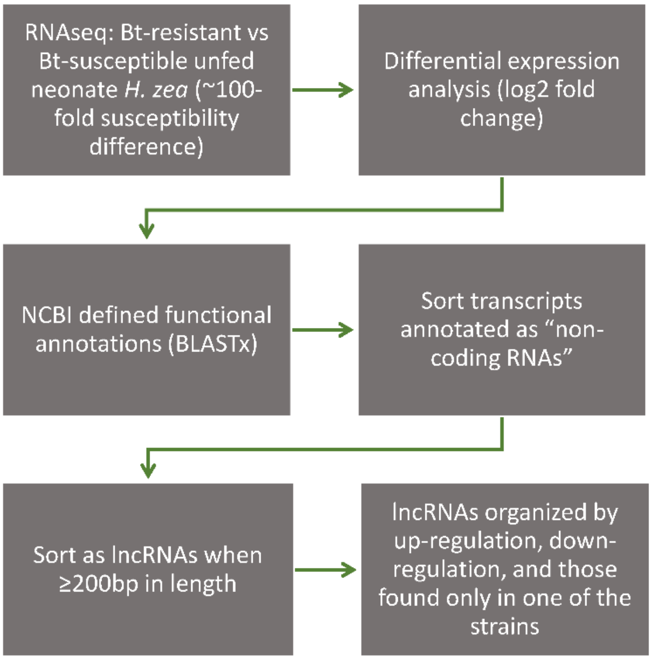

Figure S1. Workflow for identifying statistically significant, differentially expressed lncRNAs between a Bt-resistant and susceptible strain of unfed, neonate *H. zea*.

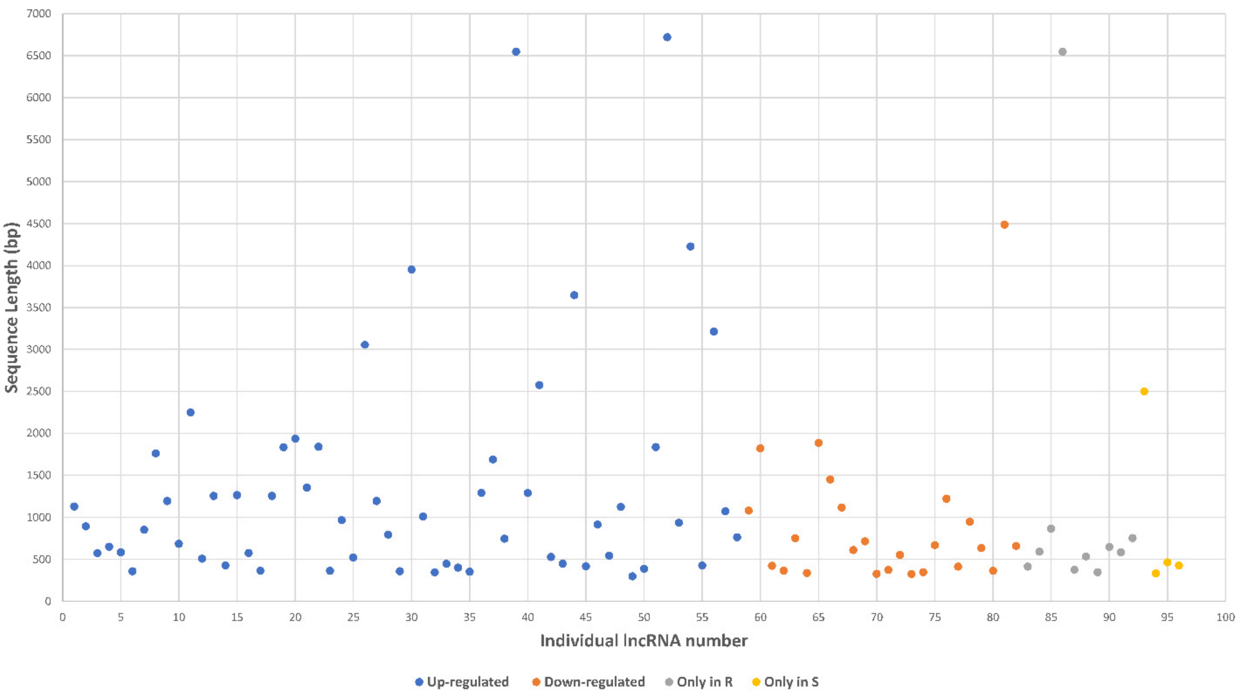

Figure S2. Sequence length in base-pairs (bp) for up-regulated and down regulated lncRNAs where a fold change could be calculated and for lncRNAs found only in the resistant (R) and susceptible (S) strains. lncRNAs were organized left to right from highest to lowest log2 fold change for each direction. LncRNAs in only the R or S categories were randomly organized. The identification numbers on the x-axis for each transcript are also shown in Supplementary Tables S1-3 along with more details about each transcript.

A

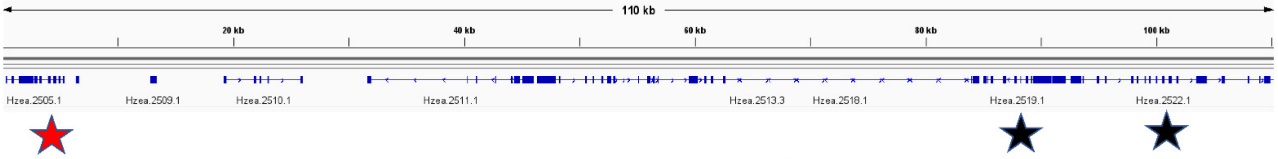

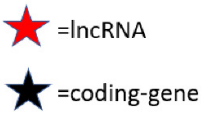

Scaffold: KZ116624.1

lncRNA ID and annotation[A]	lncRNA locus[B]	Proximal coding-gene ID[C]	Proximal transcript locus[D]	Proximal coding-gene annotation[E]	Proximal coding-gene log2 fold change[F]	BLAST alignment (% identity, E-value, Query coverage)[G]
Hzea.2506 (LOC113508874)	294-5519	Hzea.2519	85052-90956	*Helicoverpa armigera* mitochondrial import inner membrane translocase subunit TIM44 (LOC110369848), transcript variant X2, mRNA	0.334603 log2 fold increase	No significant alignment
		Hzea.2522	90996-153962	*Helicoverpa armigera* echinoderm microtubule-associated protein-like 2 (LOC110369847), transcript variant X1, mRNA	1.42313 log2 fold increase	No significant alignment

[A]lncRNA ID indicates fasta file ID number, annotation indicates NCBI defined annotation.
[B]locus number indicates base-pair coordinates for lncRNA within scaffold.
[C]coding-gene ID indicates fasta file ID number.
[D]locus number indicates base-pair coordinates for coding-gene within scaffold.
[E]coding-gene annotation indicates NCBI defined annotation.
[F]Indicates magnitude of log2 fold change and direction of expression.
[G]NCBI BLAST alignment of lncRNA to proximal coding-gene in following order: % ID, E-value, and % Query coverage.

B

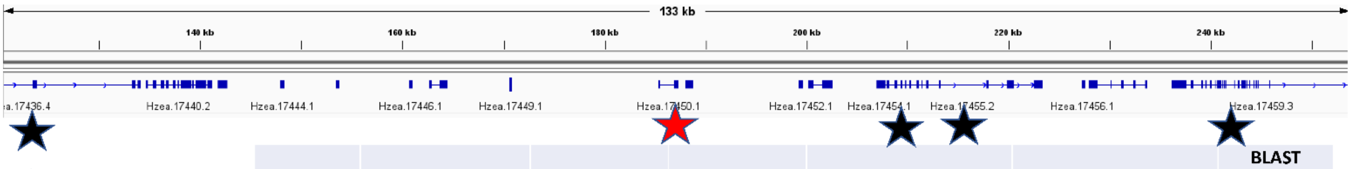

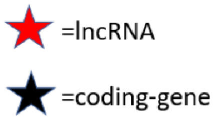

Scaffold: KZ118099.1

lncRNA ID and annotation[A]	lncRNA locus[B]	Proximal coding-gene ID[C]	Proximal transcript locus[D]	Proximal coding-gene annotation[E]	Proximal coding-gene log2 fold change[F]	BLAST alignment (% identity, E-value, Query coverage)[G]
Hzea.17450 (LOC110372550)	185452-187367	Hzea.17453	200236-202568	*Helicoverpa armigera* uncharacterized LOC110372536 (LOC110372536), transcript variant X4, mRNA	0.618834 log2 fold increase	No significant alignment
		Hzea.17436	91939-134182	*Helicoverpa armigera* CAP-Gly domain-containing linker protein 1 (LOC110372605), transcript variant X15, mRNA	0.844544 log2 fold increase	No significant alignment
		Hzea.17455	207667-223349	*Helicoverpa armigera* uncharacterized LOC110372536 (LOC110372536), transcript variant X3, mRNA	0.400524 log2 fold decrease	No significant alignment
		Hzea.17459	236185-257407	*Helicoverpa armigera* receptor-type tyrosine-protein phosphatase N2 (LOC110372540), transcript variant X3, mRNA	0.58871 log2 fold increase	No significant alignment

[A]lncRNA ID indicates fasta file ID number, annotation indicates NCBI defined annotation.
[B]locus number indicates base-pair coordinates for lncRNA within scaffold.
[C]coding-gene ID indicates fasta file ID number.
[D]locus number indicates base-pair coordinates for coding-gene within scaffold.
[E]coding-gene annotation indicates NCBI defined annotation.
[F]Indicates magnitude of log2 fold change and direction of expression.
[G]NCBI BLAST alignment of lncRNA to proximal coding-gene in following order: % ID, E-value, and % Query coverage.

C

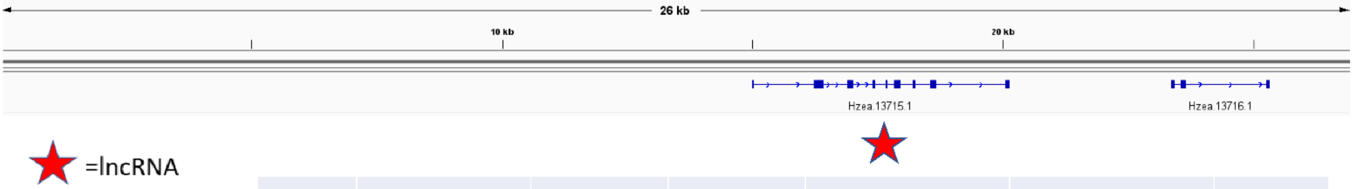

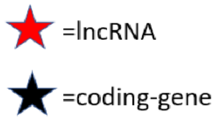

Scaffold: KZ117672.1

lncRNA ID and annotation[A]	lncRNA locus[B]	Proximal coding-gene ID[C]	Proximal transcript locus[D]	Proximal coding-gene annotation[E]	Proximal coding-gene log2 fold change[F]	BLAST alignment (% identity, E-value, Query coverage)[G]
Hzea.13715 (LOC110380503)	15001-20158	N/A	N/A	N/A	**N/A**	**N/A**

[A]lncRNA ID indicates fasta file ID number, annotation indicates NCBI defined annotation.
[B]locus number indicates base-pair coordinates for lncRNA within scaffold.
[C]coding-gene ID indicates fasta file ID number.
[D]locus number indicates base-pair coordinates for coding-gene within scaffold.
[E]coding-gene annotation indicates NCBI defined annotation.
[F]Indicates magnitude of log2 fold change and direction of expression.
[G]NCBI BLAST alignment of lncRNA to proximal coding-gene in following order: % ID, E-value, and % Query coverage.

D

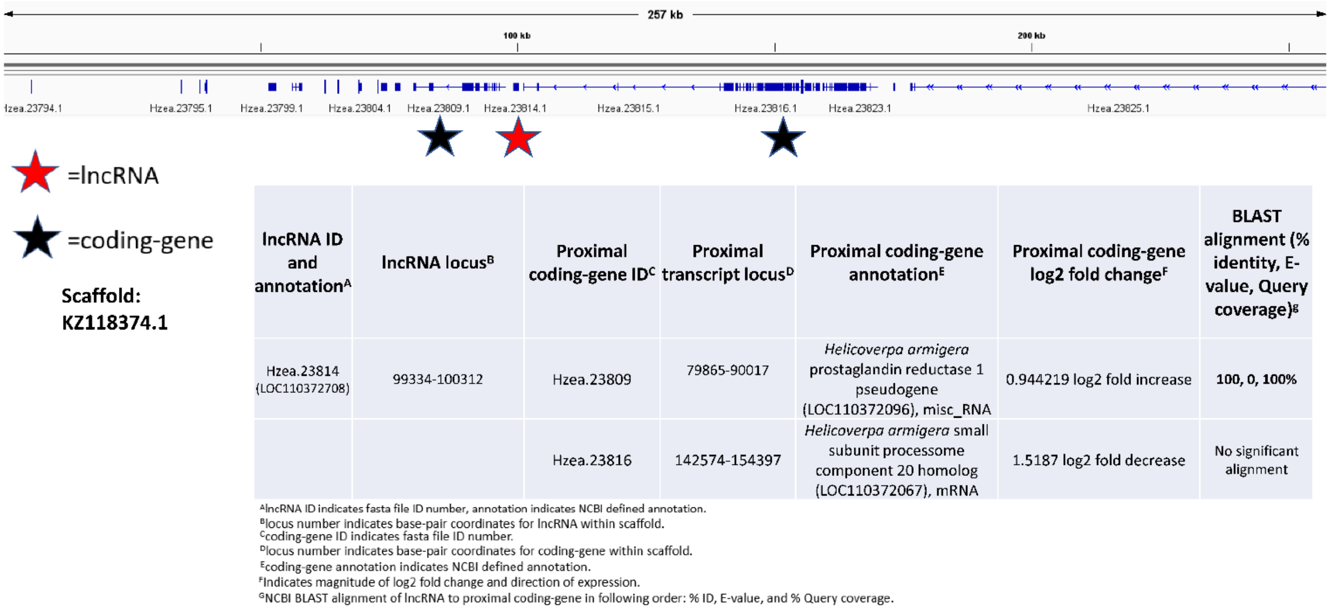

★ =lncRNA (red star)

★ =coding-gene (black star)

Scaffold: KZ118374.1

lncRNA ID and annotation[A]	lncRNA locus[B]	Proximal coding-gene ID[C]	Proximal transcript locus[D]	Proximal coding-gene annotation[E]	Proximal coding-gene log2 fold change[F]	BLAST alignment (% identity, E-value, Query coverage)[G]
Hzea.23814 (LOC110372708)	99334-100312	Hzea.23809	79865-90017	*Helicoverpa armigera* prostaglandin reductase 1 pseudogene (LOC110372096), misc_RNA	0.944219 log2 fold increase	**100, 0, 100%**
		Hzea.23816	142574-154397	*Helicoverpa armigera* small subunit processome component 20 homolog (LOC110372067), mRNA	1.5187 log2 fold decrease	No significant alignment

[A]lncRNA ID indicates fasta file ID number, annotation indicates NCBI defined annotation.
[B]locus number indicates base-pair coordinates for lncRNA within scaffold.
[C]coding-gene ID indicates fasta file ID number.
[D]locus number indicates base-pair coordinates for coding-gene within scaffold.
[E]coding-gene annotation indicates NCBI defined annotation.
[F]Indicates magnitude of log2 fold change and direction of expression.
[G]NCBI BLAST alignment of lncRNA to proximal coding-gene in following order: % ID, E-value, and % Query coverage.

Figure S3. (A-D). Genomic scaffold for up-regulated lncRNAs and identification of proximal protein coding-genes. The scaffold at the top of each A-D, depicts the range of the scaffold in kilobases (kb). The bars in blue indicate sequences present on each scaffold, with gene ID numbers below each. The red stars indicate a lncRNA, the black stars indicate a protein-coding gene. The scaffold ID number is placed directly below the legend on the left side. The table below the scaffold includes the following information about the lncRNA and coding-genes found in the scaffold from left to right: the lncRNA ID number and annotation, lncRNA loci coordinates, gene ID number of proximal coding-gene, coding-gene loci coordinates, coding-gene annotation (NCBI defined), coding-gene log2 fold change, and BLASTn alignment results (% identity, E-value, and query coverage) comparing the lncRNA and the protein coding-gene.

A

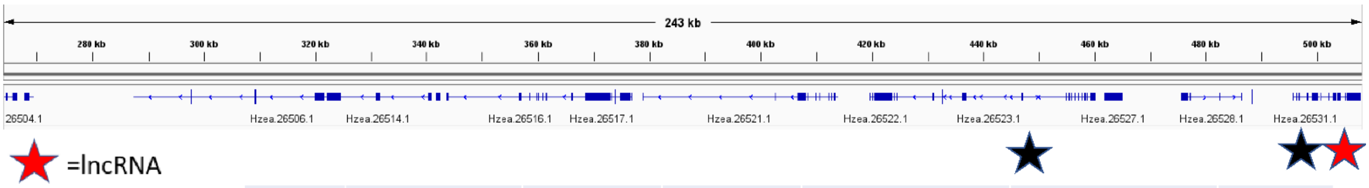

★ =lncRNA (red star)

★ =coding-gene (black star)

Scaffold: KZ118515.1

lncRNA ID and annotation[A]	lncRNA locus[B]	Proximal coding-gene ID[C]	Proximal transcript locus[D]	Proximal coding-gene annotation[E]	Proximal coding-gene log2 fold change[F]	BLAST alignment (% identity, E-value, Query coverage)[G]
Hzea.26537 (LOC110373805)	503539-507800	Hzea.26523	421869-460342	*Helicoverpa armigera* unconventional myosin-XV (LOC110372091), mRNA	0.466472 log2 fold increase	No significant alignment
		Hzea.26531	495604-500397	*Helicoverpa armigera* developmentally-regulated GTP-binding protein 2 (LOC110372053), mRNA	0.852664 log2 fold decrease	No significant alignment

[A]lncRNA ID indicates fasta file ID number, annotation indicates NCBI defined annotation.
[B]locus number indicates base-pair coordinates for lncRNA within scaffold.
[C]coding-gene ID indicates fasta file ID number.
[D]locus number indicates base-pair coordinates for coding-gene within scaffold.
[E]coding-gene annotation indicates NCBI defined annotation.
[F]Indicates magnitude of log2 fold change and direction of expression.
[G]NCBI BLAST alignment of lncRNA to proximal coding-gene in following order: % ID, E-value, and % Query coverage.

B

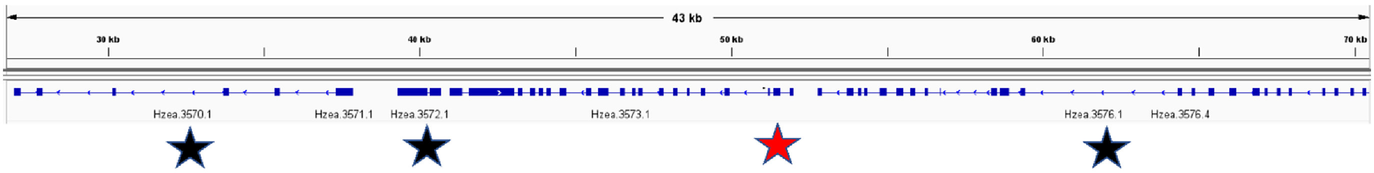

★ =lncRNA

★ =coding-gene

Scaffold: KZ116697.1

lncRNA ID and annotation[A]	lncRNA locus[B]	Proximal coding-gene ID[C]	Proximal transcript locus[D]	Proximal coding-gene annotation[E]	Proximal coding-gene log2 fold change[F]	BLAST alignment (% identity, E-value, Query coverage)[G]
Hzea.3574 (LOC110382662)	40967-52014	Hzea.3570	26998-37884	*Helicoverpa armigera* uncharacterized LOC110381452 (LOC110381452), mRNA	0.573782 log2 fold decrease	No significant alignment
		Hzea.3572	39328-40721	*Helicoverpa armigera* peptidyl-prolyl cis-trans isomerase NIMA-interacting 4 (LOC110370269), transcript variant X1, mRNA	1.58316 log2 fold decrease	No significant alignment
		Hzea.3576	52787-73597	*Helicoverpa armigera* mediator of RNA polymerase II transcription subunit 12-like (LOC110374056), transcript variant X3, mRNA	0.593304 log2 fold increase	No significant alignment

[A]lncRNA ID indicates fasta file ID number, annotation indicates NCBI defined annotation.
[B]locus number indicates base-pair coordinates for lncRNA within scaffold.
[C]coding-gene ID indicates fasta file ID number.
[D]locus number indicates base-pair coordinates for coding-gene within scaffold.
[E]coding-gene annotation indicates NCBI defined annotation.
[F]Indicates magnitude of log2 fold change and direction of expression.
[G]NCBI BLAST alignment of lncRNA to proximal coding-gene in following order: % ID, E-value, and % Query coverage.

C

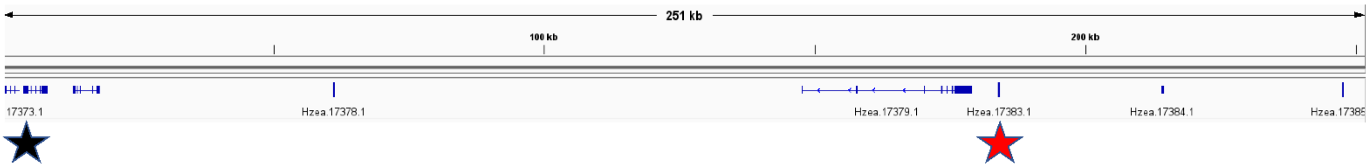

★ =lncRNA

★ =coding-gene

Scaffold: KZ118094.1

lncRNA ID and annotation[A]	lncRNA locus[B]	Proximal coding-gene ID[C]	Proximal transcript locus[D]	Proximal coding-gene annotation[E]	Proximal coding-gene log2 fold change[F]	BLAST alignment (% identity, E-value, Query coverage)[G]
Hzea.17384 (LOC110383440)	214203-214786	Hzea.17373	444-3087	*Spodoptera litura* 60S ribosomal protein L18a (LOC111357503), mRNA	0.873101 log2 fold increase	No significant alignment

[A]lncRNA ID indicates fasta file ID number, annotation indicates NCBI defined annotation.
[B]locus number indicates base-pair coordinates for lncRNA within scaffold.
[C]coding-gene ID indicates fasta file ID number.
[D]locus number indicates base-pair coordinates for coding-gene within scaffold.
[E]coding-gene annotation indicates NCBI defined annotation.
[F]Indicates magnitude of log2 fold change and direction of expression.
[G]NCBI BLAST alignment of lncRNA to proximal coding-gene in following order: % ID, E-value, and % Query coverage.

Figure S4. (A-C). Genomic scaffold for down-regulated lncRNAs and identification of proximal protein coding-genes. The scaffold in A-C depicts the range of the scaffold in kilobases (kb). The bars in blue indicate sequences present on each scaffold, with gene ID numbers below each. The red stars indicate a lncRNA, the black stars indicate a protein-coding gene. The scaffold ID number is placed directly below the legend on the left side. The table below the scaffold includes the following information about the lncRNA and coding-genes found in the scaffold from left to right: the lncRNA ID number and annotation, lncRNA loci coordinates, gene ID number of proximal coding-gene, coding-gene loci coordinates, coding-gene annotation (NCBI defined), coding-gene log2 fold change, and BLASTn alignment results (% identity, E-value, and query coverage) comparing the lncRNA and the protein coding-gene.

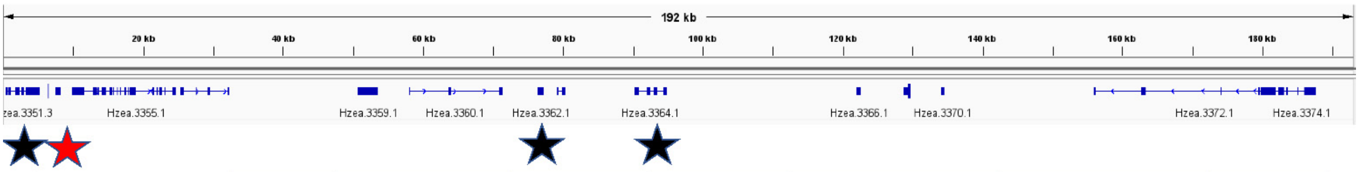

★ =lncRNA

★ =coding-gene

Scaffold: KZ116682.1

lncRNA ID and annotation[A]	lncRNA locus[B]	Proximal coding-gene ID[C]	Proximal transcript locus[D]	Proximal coding-gene annotation[E]	Proximal coding-gene log2 fold change[F]	BLAST alignment (% identity, E-value, Query coverage)[G]
Hzea.3353 (LOC110373445)	7483-8292	Hzea.3351	490-5342	*Spodoptera litura* protein groucho-like (LOC111355272), partial mRNA	0.676627 log2 fold decrease	No significant alignment
		Hzea.3362	76493-77379	Unknown Gene	1.58121 log2 fold increase	No significant alignment
		Hzea.3364	90221-94993	*Helicoverpa armigera* uncharacterized LOC110370405 (LOC110370405), mRNA	0.496072 log2 fold increase	100, 0.02, 1%

[A]lncRNA ID indicates fasta file ID number, annotation indicates NCBI defined annotation.
[B]locus number indicates base-pair coordinates for lncRNA within scaffold.
[C]coding-gene ID indicates fasta file ID number.
[D]locus number indicates base-pair coordinates for coding-gene within scaffold.
[E]coding-gene annotation indicates NCBI defined annotation.
[F]Indicates magnitude of log2 fold change and direction of expression.
[G]NCBI BLAST alignment of lncRNA to proximal coding-gene in following order: % ID, E-value, and % Query coverage.

Figure S5. Genomic scaffold for lncRNAs found only in the R strain and identification of proximal protein coding-genes. The scaffold at the top depicts the range of the scaffold in kilobases (kb). The bars in blue indicate sequences present on each scaffold, with gene ID numbers below each. The red stars indicate a lncRNA, the black stars indicate a protein-coding gene. The scaffold ID number is placed directly below the legend on the left side. The table below the scaffold includes the following information about the lncRNA and coding-genes found in the scaffold from left to right: the lncRNA ID number and annotation, lncRNA loci coordinates, gene ID number of proximal coding-gene, coding-gene loci coordinates, coding-gene annotation (NCBI defined), coding-gene log2 fold change, and BLASTn alignment results (% identity, E-value, and query coverage) comparing the lncRNA and the protein coding-gene.

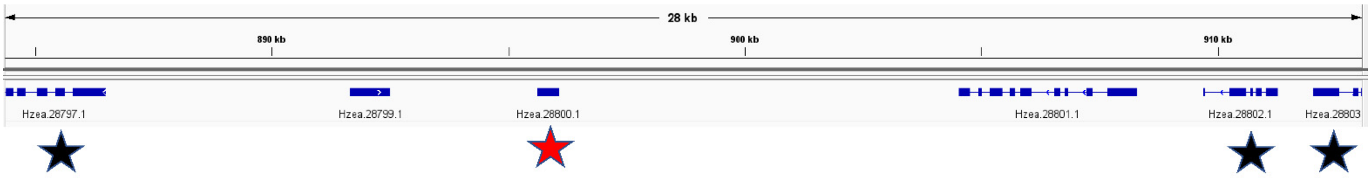

★ =lncRNA

★ =coding-gene

Scaffold: KZ118643.1

lncRNA ID and annotation[A]	lncRNA locus[B]	Proximal coding-gene ID[C]	Proximal transcript locus[D]	Proximal coding-gene annotation[E]	Proximal coding-gene log2 fold change[F]	BLAST alignment (% identity, E-value, Query coverage)[G]
Hzea.28800 (LOC113495312)	895622-896089	Hzea.28797	883429-886507	*Helicoverpa armigera* putative ATPase N2B (LOC110384128), mRNA	0.321567 log2 fold increase	No significant alignment
		Hzea.28802	909695-911283	*Helicoverpa armigera* prothoracicotropic hormone-like (LOC110384160), mRNA	1.44582 log2 fold increase	No significant alignment
		Hzea.28803	912030-915696	*Helicoverpa armigera* zinc finger protein 239-like (LOC110384141), transcript variant X1, mRNA	0.729596 log2 fold increase	No significant alignment

[A]lncRNA ID indicates fasta file ID number, annotation indicates NCBI defined annotation.
[B]locus number indicates base-pair coordinates for lncRNA within scaffold.
[C]coding-gene ID indicates fasta file ID number.
[D]locus number indicates base-pair coordinates for coding-gene within scaffold.
[E]coding-gene annotation indicates NCBI defined annotation.
[F]Indicates magnitude of log2 fold change and direction of expression.
[G]NCBI BLAST alignment of lncRNA to proximal coding-gene in following order: % ID, E-value, and % Query coverage.

Figure S6. Genomic scaffold for lncRNAs found only in the S strain and identification of proximal protein coding-genes. The scaffold at the top depicts the range of the scaffold in kilobases (kb). The bars in blue indicate sequences present on each scaffold, with gene ID numbers below each. The red stars indicate a lncRNA, the black stars indicate a protein-coding gene. The scaffold ID number is placed directly below the legend on the left side. The table below the scaffold includes the following information about the lncRNA and coding-genes found in the scaffold from left to right: the lncRNA ID number and annotation, lncRNA loci coordinates, gene ID number of proximal coding-gene, coding-gene loci coordinates, coding-gene annotation (NCBI defined), coding-gene log2 fold change, and BLASTn alignment results (% identity, E-value, and query coverage) comparing the lncRNA and the protein coding-gene.
